# Incongruity of LVH regression with persistent inopportune diastolic dysfunction; results following AVR for severe aortic stenosis. Sponsored by the American Heart Association

**DOI:** 10.1186/1532-429X-11-S1-P94

**Published:** 2009-01-28

**Authors:** Robert WW Biederman, Ronald B Williams, Saundra B Grant, Wadih Nadour, Vikas K Rathi, Diane A Vido, June Yamrozik, Geetha Rayarao, James A Magovern, Mark Doyle

**Affiliations:** Allegheny General Hospital, The Gerald McGinnis Cardiovascular Institute, Pittsburgh, PA USA

**Keywords:** Aortic Stenosis, Diastolic Function, Aortic Valve Replacement, Deceleration Time, Pressure Overload

## Background

Very elegant invasive animal and human studies have demonstrated that in subjects with pressure overload due to severe aortic stenosis (AS), following aortic valve replacement (AVR), while myocyte regression is quite rapid, interstitial collagen content regression lags.

We **hypothesize** that this discord manifests as a thwarted improvement in diastolic function as related to LVH regression after AVR for AS.

## Methods

**Figure 1:**
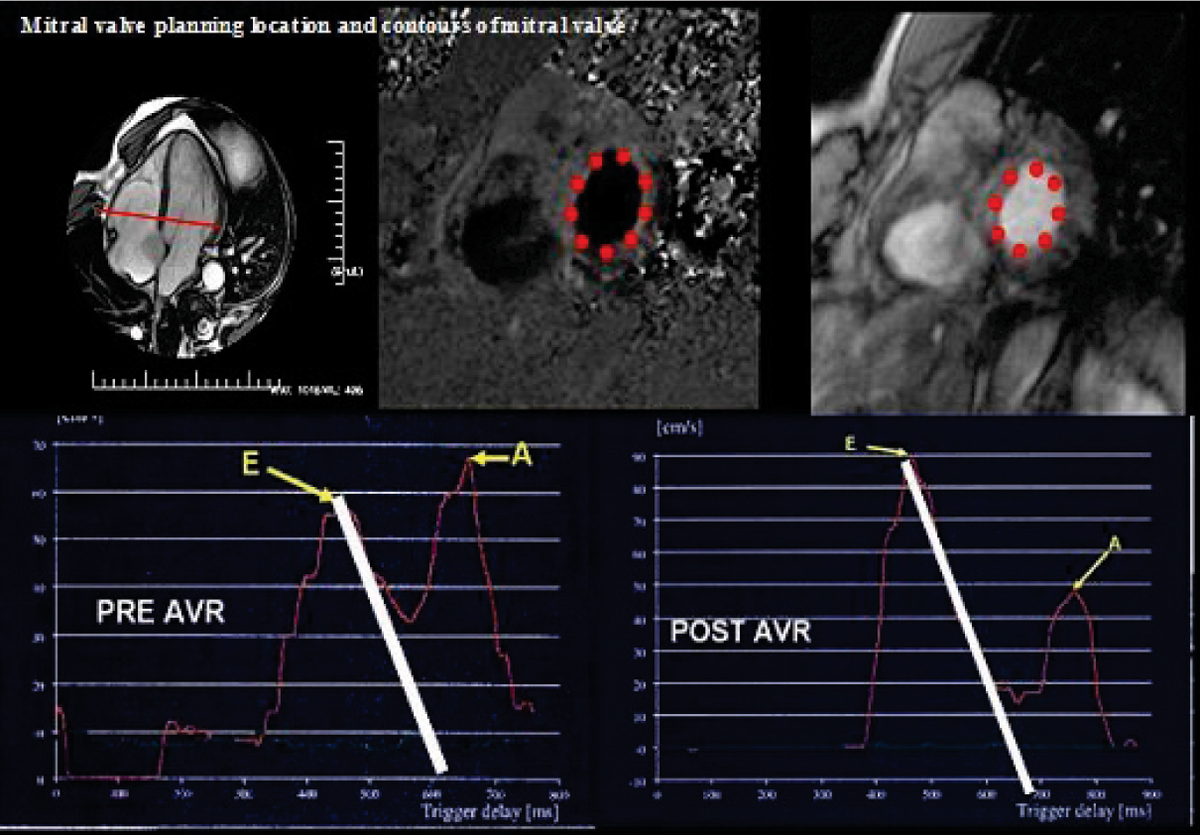
Figure 1

Ten patients with severe, but compensated AS underwent 4 serial 3D cardiac MRIs (CMR) with 1.5 T EXCITE (GE Milwaukee, WI) out to 4 years (40 time-points: baseline, 6 months, 1 year and up to 4 years). 3D LV volumetrics were measured. LV diastolic function was assessed by a phase velocity mapping slice placed at the tips of the mitral valve leaflets, acquired in a through-plane manner with temporal resolution of 25 ± 5 ms. Interrogation of resultant time-velocity curves was performed to resolve: 1) E:A ratio as mean and peak absolute and relative velocities 2) deceleration time. Figure 1.

## Results

All patients survived AVR and were available for serial follow-up over 4 time periods (mean 3 ± 1) out to 4 years. E:A ratios and/or morphology almost uniformly improved (9/10 pts) from 0.9:1 to 1.7:1 (p < 0.005), including 4 patients improving one complete grade. This was moderately well correlated with LV mass index regression (r = 0.55, p = < 0.05). Deceleration time also improved (233 vs. 192 ms, p < 0.005). While EF improved (55 ± 22 to 65 ± 11%, p < 0.05), as did LV geometry (1.07 ± 0.2 to 0.94 ± 0.24 g/m^2^, p < 0.05), neither were tightly correlated with improvements in diastolic function. However, using predictive modeling from historic controls, the improvement seen in diastolic function was incomplete and tardy. Specifically, while there were 4 patients that returned to normal diastolic function as per morphologic criteria, even these patients did not return to pre-morbid e:a ratios or deceleration times of the normal historic cohort. It followed that the remaining patients, except for one, improved only in arithmatic metrics. Importantly, while the LVH regresssed quickly, most occured within the first 6 months, there was a lag in diastolic function improvement by >12 months.

## Conclusion

Following AVR for severe AS, as expected, marked improvements in LV mass regression occur; however, improvements in diastolic physiology are incomplete and quite tardy, not paralleling structural changes. Pathophysiologically, it is likely that the **interstitium**, failing to be as heavily modulated by relief of pressure overload as are the **myocytes**, contributes to a paradoxical *increase* in diastolic stiffness. This interplay between collagen content and myocyte results in a less than expected improvement in myocardial diastolic properties following afterload relief contributing to residual dyspnea.

